# Screening TLR4 Binding Peptide from *Naja atra* Venom Glands Based on Phage Display

**DOI:** 10.3390/toxins16030113

**Published:** 2024-02-24

**Authors:** Runhan Li, Yezhong Tang, Zening Chen, Yang Liu

**Affiliations:** 1 Key Laboratory of Ecology of Rare and Endangered Species and Environmental Protection, Guangxi Normal University, Ministry of Education, Guilin 541006, China; lirunhan@stu.gxnu.edu.cn; 2 Chengdu Institute of Biology, Chinese Academy of Sciences, Chengdu 610041, China; tangyz@cib.ac.cn

**Keywords:** TLR4, snake venom gland, phage display, surface plasmon resonance (SPR), *Naja atra*

## Abstract

Toll-like receptor 4 (TLR4) is a crucial inflammatory signaling pathway that can serve as a potential treatment target for various disorders. A number of inhibitors have been developed for the TLR4 pathway, and although no inhibitors have been approved for clinical use, most have been screened against the TLR4-MD2 conformation. The venom gland is the organ of venomous snakes that secretes substances that are toxic to other animals. The level of gene transcription in venom glands is different from that in other tissues, includes a large number of biologically active ingredients, and is an important natural resource for the development of new drugs. We constructed a T7 phage display library using the cobra (*Naja atra*) venom gland from the Guangdong Snake Breeding Plant and performed three rounds of screening with TLR4 as the target, randomly selecting monoclonal phage spots for PCR followed by Sanger sequencing. The obtained sequences were subjected to length analysis, molecular docking, solubility prediction, and stability prediction, and a peptide containing 39 amino acids (NA39) was finally screened out. The BLAST results indicated that NA39 was a sequence in RPL19 (Ribosomal Protein L19). After peptide synthesis, the binding ability of NA39 to TLR4 was verified by the surface plasmon resonance (SPR) technique. In this study, a new peptide that can specifically bind TLR4 was successfully screened from the cobra venom gland cDNA library, further demonstrating the effectiveness of phage display technology in the field of drug discovery.

## 1. Introduction

TLR4 is a related protein in the Toll-like receptor (TLR) family of innate immune pattern recognition receptors, which is expressed in myeloid cells, microglia, astrocytes, and peripheral primary sensory cells [1]. Numerous studies have shown that TLR4 is involved in the occurrence of many diseases, the excitation of neurons, glial cells, and inflammatory cells, and plays an important role in the formation and maintenance of inflammation, pain, and cancer [2,3,4,5,6,7]. Targeting TLR4 can relieve many types of pain, including inflammatory pain, neuropathic pain, and post-illness pain [8,9,10].

Blocking TLR4 activation is an effective approach to managing TLR4-mediated diseases. At present, the research on analgesic drugs based on TLR4 is still in the early stages. The structure of TLR4 consists of three parts: extracellular, transmembrane, and intracellular. The extracellular part is a repetitive leucine sequence, which mediates the recognition of pathogen-related molecules, and the intracellular part has homology with the intracellular fragment of the interleukin-1 receptor [11]. The extracellular domain of TLR4 forms a heterodimer with myeloid differentiation protein 2 (MD2), while binding with ligands (such as lipopolysaccharide (LPS)), two TLR4-MD2 complexes combine to form a homodimer. The latter leads to the polymerization of the intracellular domain and then activates the downstream reaction, initiating the downstream channels through the MyD88-dependent and -independent pathways to promote the expression of inflammatory cytokines and chemokines [12]. There exist three potential ways to prevent the TLR4 signaling pathway from being activated. Firstly, by inhibiting dimerization between TLR4 and MD2, secondly, by preventing trimer formation between TLR4-MD2 and LPS, and thirdly, by stopping dimerization of the two trimers. Most research has focused on inhibiting the binding of the TLR-MD2 complex to LPS; however, no pharmaceutical interventions have successfully completed clinical trials at present. Current studies reported a few molecules that specifically bind to the extracellular domain of TLR4 in the absence of polypeptide molecules [13,14,15,16,17]. The structure of the TLR4 extracellular domain, appearing as a horseshoe shape, is relatively stable, probably due to its leucine-rich repeating unit, which results in a large protein molecule. Binding ligands can generate relatively stable molecular patterns and thus have great potential in the research and development of clinical drugs.

Venom glands produce toxins through complex and delicate mechanisms and are an important glandular organ of toxin-producing organisms. Studies have shown that the level of gene transcription in venom glands is different from that in other tissues and can efficiently and accurately characterize various complex toxin components and regulate the production and release of venom [18]. Due to the complexity of the process of cDNA library translation, we use the information on the cobra venom gland transcription to obtain a large number of other new protein molecules and peptide fragments that may have biological activity in addition to venom component substances. It can be seen that screening active components from the cobra venom gland cDNA library is an effective way to develop new active molecules. Venom gland tissue contains many genes that are specific to conventional tissue expression, and these genes are mainly characterized by a large number of proteins and peptides, making them a perfect reservoir of naturally occurring active molecules for the development of natural medicines.

Phage display technology proves to be a highly effective screening tool, as it enables the efficient identification of phages capable of binding to specific target molecules from a vast pool of phages, owing to the inherent intermolecular interactions involved [19]. Phage display technology has been developed to a relatively mature stage, and a variety of vectors are available and widely used in new vaccine development, new drug research and development [20], disease treatment and diagnosis [21], and so on. Through the T7 phage, exogenous genes can be inserted into the C-terminal of the 10B locus, which is then expressed on the phage surface. The T7 phage is very stable in structure and can remain highly active under the conditions of high salt, denaturant, and extreme pH [22]. In this study, the T7 phage was selected as the carrier for the construction of a phage display library. The library underwent screening on an ELISA plate that was coated with TLR4, serving as the designated target protein. The phage capable of binding to the target protein was selectively enriched, allowing for the subsequent determination of the target polypeptide sequence using sequencing. 

There is usually a false positive result from biological elutriation [23]. In order to improve the efficiency of screening, we combined bioinformatics databases and computer algorithms, such as PSIPRED 4 (Protein Structure Prediction Server) for predicting the secondary structure of peptides [24], SWISS-MODEL [25] for predicting the tertiary structure of peptides, and the HDOCK server [26] for predicting the molecular docking of TLR4 with the peptides. The peptide with the largest binding was selected.

Surface plasmon resonance (SPR) is used in the field of biology to investigate molecular interactions. By leveraging the principles of reflection spectroscopy, the dynamic process of molecular interaction can be efficiently and sensitively determined by monitoring the angular displacement of resonance. Additionally, the affinity constant of the ligand-receptor interaction can be quantified [27]. The binding and dissociation signals between corresponding peptides and TLR4 were examined using SPR technology. The observed changes in signal intensity with varying concentrations suggested the presence of a novel polypeptide capable of specific binding to TLR4 from the phage display library of the cobra venom gland.

The purpose of this study was to screen out a new binding peptide of TLR4 from the cobra venom gland cDNA library by phage display, which is expected to be applied to the research of TLR4-related physiological functions and drug development, aiming to provide inspiration and help for future research in related fields.

## 2. Results

### 2.1. Construction of the T7 Phage Display Library of the Cobra Venom Gland

**Figure 1:**
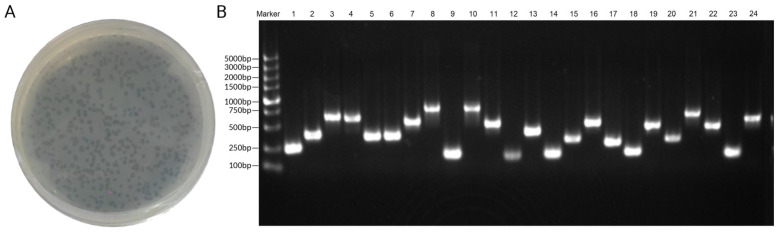
Verification of the phage library titer. (**A**). Each transparent dot in the plate (10^12^) represents a monoclonal phage, and the titer of the phage library can be calculated according to the number of monoclonal phages. (**B**). Monoclonal clones in the phage library were selected for PCR, and gel electrophoresis showed that gene fragments with different lengths were successfully inserted into the phages.

It was verified that the titer of the phage-amplified library was 2.3 × 10^13^ pfu/mL (Figure 1A). Twenty-four monoclonal colonies were randomly selected from the library for PCR, and 5 μL of PCR products were electrophoresed in 1% agarose gel for 15–20 min (Figure 1B). The results of electrophoresis showed that the sequences of fragments with different sizes were successfully inserted into the phages, and the recombination rate of the phage display library was 76.92% after sequencing. The constructed phage display library was suitable for subsequent panning experiments.

### 2.2. Biopanning

**Figure 2:**
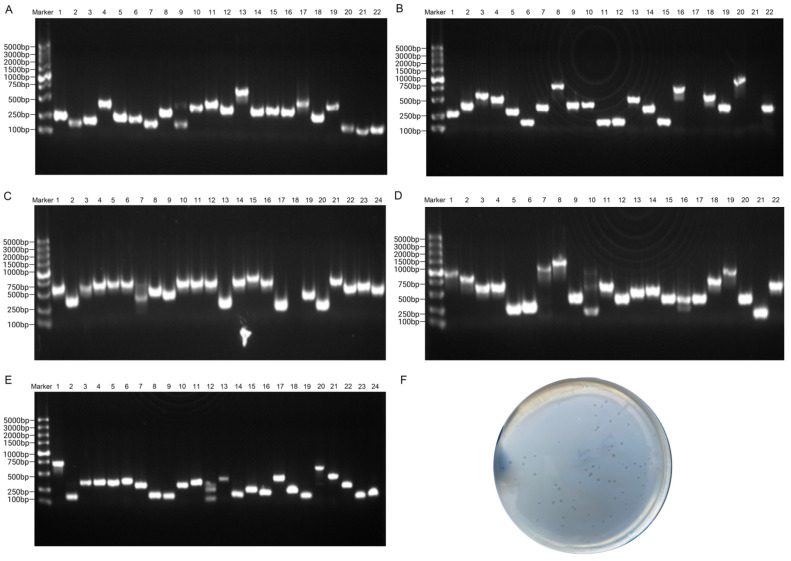
Phage screening results. (**A**–**E**). PCR gel electrophoresis patterns of 114 monoclonal phages (10^17^) that were randomly selected. (**F**). The culture plate with a dilution concentration of 10^17^.

TLR4 was chosen as the target to screen the phage library for three rounds. The phage extraction buffer collected at the end of each round of screening was added to a fresh bacterial solution of BLT5403 with an OD value of 0.5–0.6, and amplification was incubated at 37 °C with shaking until the bacterial solution was clear from a turbid state. The titer of the screened library after three rounds of amplification culture was 9.2 × 10^18^ pfu/mL after the dilution of culture. It was determined that the culture plate with a dilution concentration of 10^17^ (Figure 2F) was suitable for picking single colonies for PCR. A total of 114 (Figure 2A–E) single colonies were selected for PCR, and 5 μL of PCR products were electrophoresed in a 1% agarose gel at 180 V for 15–20 min to obtain clear bright bands.

### 2.3. Molecular Docking Screening Potential Peptides

Randomly selected monoclonal phage spots were selected for PCR and then Sanger sequencing, resulting in 109 sequences. Sequence identity alignment of the sequencing results was performed using bioedit software [28], where 21 sequences had a similarity greater than 0.8, and 88 sequences remained. BLAST analysis of the connotation sequence using the NCBI database confirmed that the homologous species were snakes, including *Notechis scutatus*, *Pseudonaja textili*, *Sinomicrurus macclellandi*, *Thamnophis sirtalis*, and so on. According to the properties and differences between proteins and polypeptides, the base sequence lengths of less than 500 bp were retained. After the base sequences were translated into amino acid sequences, the translation results of the +1 reading frame were retained for subsequent analysis, while the sequences with a stop codon in the first ten amino acids were removed. Finally, 42 effective sequences were kept. Based on the amino acid sequences, the binding scores with TLR4 protein were predicted by the HDOCK Server, while the solubility was predicted by the Protine-Sol, and the stability was predicted by the Expasy-ProtParam tool for 42 sequences. We listed information for the top 10 sequences (Table 1), where a molecular docking confidence score of more than 0.7 indicates that two molecules have good binding, a solubility prediction score of more than 0.45 indicates that the predicted molecule has good solubility, and an instability score of less than 40 indicates that the predicted molecule has good stability. The evaluation process involved a thorough assessment of the top 10 sequences, ultimately leading to the selection of NA39 (with a binding score of 0.9172, a solubility score of 0.701, and an instability score of 39.85) for further validation. This decision was based on a set of criteria, including a maximum amino acid count of 45, a docking score above 0.9, an instability score below 40, and a preference for higher solubility. It showed great potential to bind to the TLR4 protein and had promising solubility and stability.

The base sequence of peptide NA39 was 5′-AAT TCG GGC AGG CAT ATG TAC CAT AGC TTG TAC TTG AAA GTA AAA GGT AAT GTG TTC AAG AAC AAG CGC ATT CTG ATG GAG CAC ATC CAT AAA CTG AAG GCA GAT AAG GCT CGC AAG-3′ (Supplemental File S1). The Blastn alignment results showed that the base sequence of peptide NA39 was a fragment of mRNA of ribosomal protein L19 (RPL19) in Eastern Brownsnake (*Pseudonaja textilis*); the Sequence ID was XM_026717514.1, the matching range was 433–542, the matching degree was 126 bits (68%), 108/110 (98%), and the interval was 0/110 (0%), indicating that the source of peptide NA39 sequence was indeed the mRNA of cobra species. The results of the Blastx alignment showed that the amino acid sequence translated from the +1 reading frame of the mRNA sequence was N′-NSGRHMYHSLYLKVKGNVFKNKRILMEHIHKLKADKARK-C′. It is a fragment of the 60 s ribosomal protein L19 of *Naja Naja*; the Sequence ID was KAG8139942.1, the matching range was 97–132, the matching degree was 75.9 bits (185), 36/36 (100%), and the interval was 0/36 (0%). These results indicate that peptide NA39 is a small fragment of a natural polypeptide in the native protein. 

### 2.4. Docking Results of NA39 and the TLR4 Molecule

**Figure 3:**
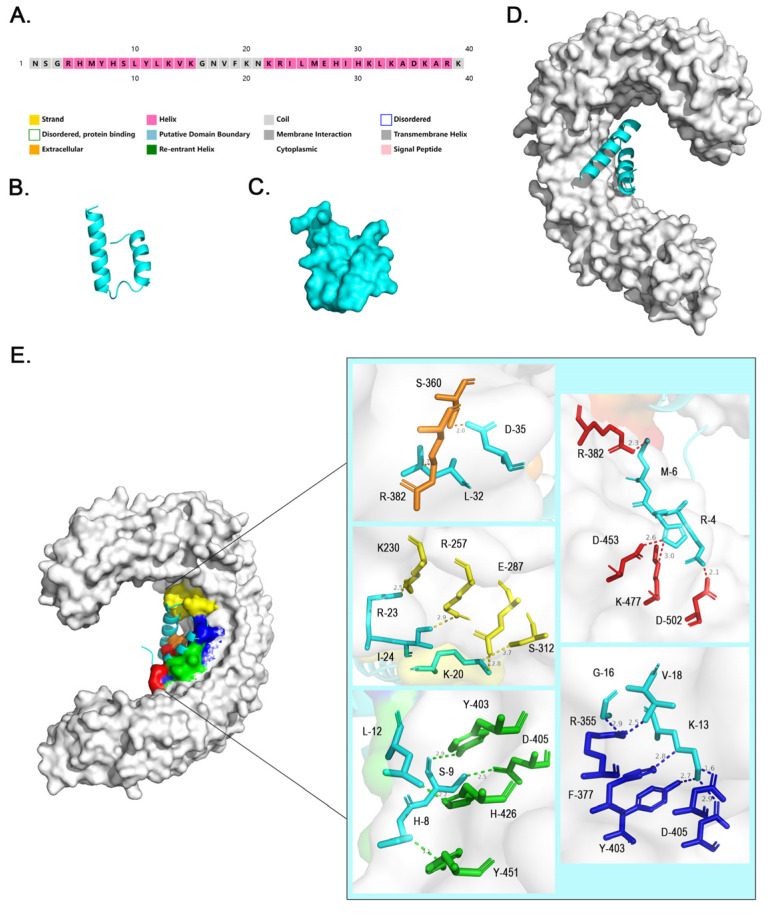
Structural diagram of NA39 and schematic diagram of the docking results to the TLR4 molecule. (**A**): Diagram of the predicted secondary structure of NA39 using PSIPRED 4.0, where the pink part indicates the alpha helix. (**B**): Predicted tertiary structure model of NA39 using SWISS-MODEL, cartoon format. (**C**): Predicted tertiary structure model of NA39 using SWISS-MODEL, surface format. (**D**): Binding diagram of TLR4 and NA39; the grey part is TLR4 in surface format, and the cyan part is NA39 in cartoon format. (**E**): Binding map of TLR4 and NA39, the large figure on the left is the binding map, where different colored blocks indicate different regions of the binding site, and the small figure on the right is the enlargement of the regions of different colored blocks, showing all binding sites that act at a distance of less than 3Å and overlap with TLR4-MD2. The key residues of the TLR4 part are colored orange, yellow, green, red, and dark blue. The key residues of NA39 are colored cyan.

When using the HDOCK Server for molecular docking, we used the extracellular portion of the TLR4 protein and the amino acid sequence of NA39 provided by the PDB number 3fxi_A file. After the HDOCK Server created the molecular docking prediction, 100 docking prediction results were obtained. According to the score ranking, the group with the highest score was selected, and the results were visualized by PyMol (Figure 3) according to the molecular binding sites. The docking results showed that there was probably a reliable binding between the TLR4 protein and NA39 (Supplemental File S2: the NA39 and NA39-TLR4 PDB files).

### 2.5. Synthetic Peptide NA39

**Figure 4:**
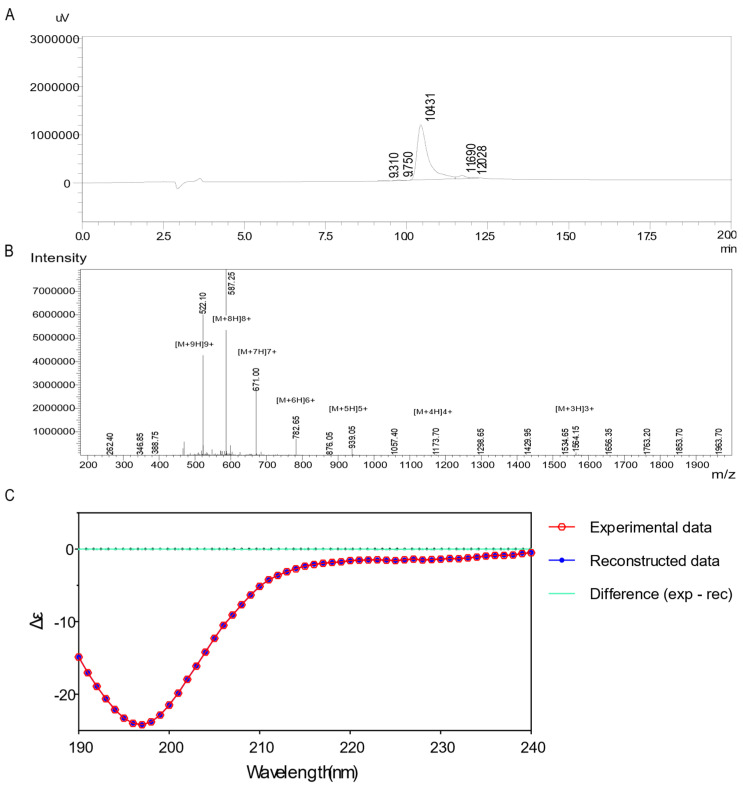
Detection and validation of synthetic NA39. (**A**). HPLC assay: the result showed that the purity of synthetic NA39 was 95.192%. (**B**). MS assay: the result showed that the maximum charge-to-mass ratio of NA39 was [M + 9H]^9+^, and the molecular weight of NA39 could be calculated from the average charge-to-mass ratio as 4690, which matched the theoretical molecular weight results of the molecular weight calculation of the amino acid alone. (**C**). CD spectra of the polypeptide: NA39 circular dichroism detection showed that the primary secondary structure of the synthesized NA39 was helix, which matched with the secondary structure prediction result (Figure 3A).

Based on the sum of the molecular weights of each individual amino acid comprising NA39 minus the molecular weight of the peptide bond forming dehydration, the theoretical molecular weight size of NA39 was finally calculated to be 4690.576 Da. Peptide NA39 was synthesized in dry powder form using the solid phase synthesis Fmoc method (SPPS) and validated by quantitative and qualitative analysis using mass spectrometry (MS) and high-performance liquid chromatography (HPLC). The purity determined by HPLC was 95.192% (Figure 4A). The charge-mass ratios of [M + 9H]^9+^, [M + 8H]^8+^, [M + 7H]^7+^, [M + 6H]^6+^, [M + 5H]^5+^, [M + 4H]^4+^, and [M + 3H]^3+^ were 522.10, 587.25, 671.00, 782.65, 939.05, 1173.70, and 1564.15 Da, respectively. According to the highest peak [M + 8H]^8+^, the molecular weight of NA39 was calculated as follows:

(1) $$MW=\frac{m}{z}\times C-C$$

where MW is the molecular mass, *m* is the mass of the molecular ion peak, *z* is the ion charge number, and *C* is the charge number of the positive ionization reagent. The mass spectrometry results showed that the molecular weight of the synthesized NA39 was consistent with the total molecular weight we calculated from the molecular weights of individual amino acids, indicating that the synthesized NA39 was reliable (Figure 4B). The solubility of peptide NA39 was 76.9 mg/mL, which was in the range of 1–10 g/mL, indicating that peptide NA39 was easy to dissolve and suitable for subsequent SPR experiments. The circular dichroism (CD) integration of synthetic NA39 was fitted by the CDSSTR method in DichroWeb to obtain the following results (Figure 4C): NRMSD: 0.001 (less than or equal to 0.01 is a good fitting result); helix segments per 100 residues: 3.706; average helix length per segment: 14.839; strand segments per 100 residues: 4.141; average strand length per segment: 3.560; average strand length per segment: 3.560; and Helix1 (0.4), Helix2 (0.15), Strand1 (0.06), Strand2 (0.08), Turns (0.1), Disordered (0.2), Total (0.99). The CD fitting results indicated that the primary secondary structure unit of synthetic NA39 was α-helix, which was basically consistent with the secondary structure prediction results (Figure 3A).

### 2.6. SPR Verification of the Binding Force between Peptide NA39 and TLR4

**Figure 5:**
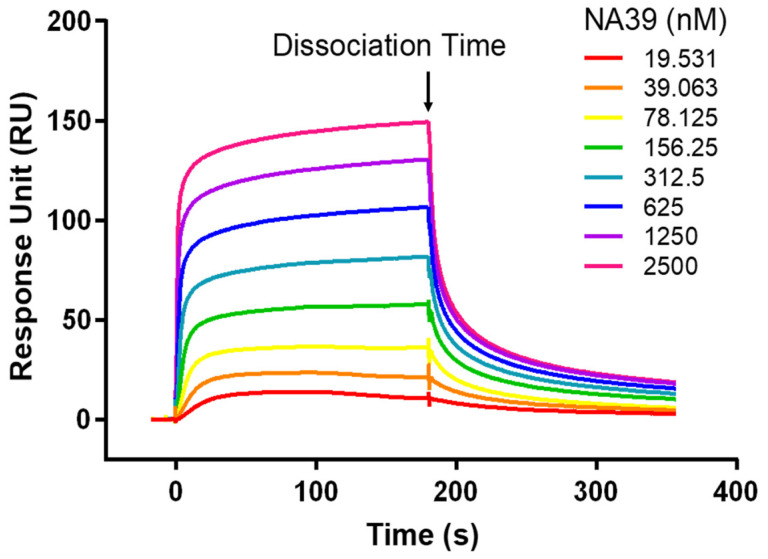
The binding force between peptide NA39 and TLR4 measured with SPR. Different colors in the SPR results represent different concentrations. The sensing map showed that different concentrations of peptide NA39 and TLR4 can respond, and the response results were proportional to the concentration. Dissociation began at 180 s, and all results were obtained by subtracting the blank group from the experimental group.

SPR was used to verify whether peptide NA39 and TLR4 could bind effectively. TLR4 was coupled to the CM5 chip, and the concentration gradients of peptide NA39 were set to 0 nM, 19.53125 nM, 39.0625 nM, 78.125 nM, 156.25 nM, 312.5 nM, 625 nM, 1250 nM, and 2500 nM, respectively, and the peptide with different concentrations flowed through the chip in descending order. The results showed that peptide NA39 could bind effectively to the TLR4 protein in a significant concentration-dependent manner (Figure 5). Biacore Evaluation Software 3.2 was used to analyze the binding curves. The equilibrium dissociation constant (KD) of peptide NA39 binding to TLR4 was 3.38 × 10^−7^ M based on affinity fitting, with smaller KD values representing more powerful interactions. The KD value of peptide NA39 was from 10^−8^ M to 10^−7^ M, indicating that peptide NA39 and TLR4 could bind well [29].

## 3. Discussion

In the TLR4 pathway, the utilization of antagonists that specifically target the TLR4-MD2 complex has been observed to yield notable analgesic effects [30]; however, antagonists targeting the TLR4-MD2 complex have higher structural requirements, which makes it more difficult to design and synthesize drugs. These drugs may also interfere with the normal immune response and have other unknown effects on the human immune system [31]. Antagonists that directly bind the extracellular part of TLR4 will be more targeted and have a relatively limited effect by only affecting the binding area, which can relatively avoid some side effects.

The therapeutic potential of small-molecule compounds or antibodies targeting TLR4 has been evaluated in previous studies. According to the process of TLR4 signaling pathway activation, it is known that dimer formation between TLR4 and MD2 is an indispensable part. Compounds encompassing pyrimidine guanidine scaffolds have been observed to establish hydrogen bonds with TLR4 via guanidine groups, thereby demonstrating favorable antagonistic activity in cellular systems [13,14]. Nevertheless, these compounds manifest cytotoxicity at concentrations of 20 mM. Consequently, enhancing the efficacy and drug-like characteristics of these compounds constitutes the central objective for forthcoming investigations. The administration of Crocin, a distinctive carotenoid that dissolves in water, exhibited a notable reduction in the upregulation of TLR4 mRNA expression and protein levels induced by cisplatin. Molecular docking analysis revealed that Crocin potentially exerts its effects through binding to TLR4 [15]. Ferulic acid inhibits LPS-induced neuroinflammation using JNK/NFKB and TLR4 inhibition, as reported by Rehman et al. [16]. In docking simulations, FA inhibited downstream inflammatory signaling by interfering with the TLR4 complex. The binding sites of the above small-molecule compounds with TLR4 are all within the binding range of MD2 and TLR4. Monoclonal antibodies targeting TLR4 monomers, such as human anti-TLR4 IgG2, have been documented to inhibit NF-κB activation in cells expressing LPS-induced TLR4. It is postulated that this antibody may impede the interaction between MD2 and TLR4, thereby obstructing the transduction of the LPS-induced TLR4 signaling pathway [17]. These investigations suggest that the exploration of regulatory molecules that target TLR4 monomers represents a promising avenue for the advancement of the TLR4 signaling pathway. 

Peptides, as natural ligands of various receptors and channels in organisms, provide an ideal substance for developing new drugs targeting specific receptors. Peptide drugs have the advantages of high efficacy and low toxicity. In recent years, the number of peptide drugs approved by the FDA has increased year by year, with broad development prospects [32]. Natural peptide medication research and development using the traditional separation approach is time- and resource-intensive. Phage display technology breaks the limitation of requiring prior conditions, such as known peptide sequence and structure [33], and can directly screen unknown polypeptide ligands with high affinity and biological activity through biological panning [34], which provided an effective experimental scheme for screening peptides specifically binding to TLR4 in this study.

As an important organ of the cobra, in addition to the general function of organs to maintain growth and development and the special function of producing and releasing venom, the venom gland also has a series of mechanisms to protect itself from toxins [35]. The phage display library constructed using the cDNA library of the cobra venom gland can display most of the substances represented by the transcriptome of the venom gland, including toxin components, physiological activity regulating substances, and substances that protect against toxin invasion. Therefore, the phage display library we constructed can screen new substances with a variety of medicinal potentials using different targets. It can also be used to develop targets related to the function of the venom gland, providing more clues for the development of medicinal targets for other homologous organs, and to develop antivenom drugs.

In this study, a polypeptide binding for the TLR4 monomer may have been developed for the first time. A comparison of the binding site characteristics between TLR4-NA39 and TLR4-MD2 was conducted. Our findings indicate that there is a significant overlap in the amino acids involved in the binding of NA39 to TLR4 (specifically within the range of 230–526) and MD2 to TLR4 (specifically within the range of 15–313). Notably, amino acid 312 serves as the site of overlap between these two binding interactions. This might hinder the binding of MD2 to TLR4 to some extent, reducing the binding efficiency, and further influencing the activation of the TLR4 pathway. The effects of NA39 in cell and animal models require further investigation. 

It is generally believed that short peptides have better tissue permeability and lower immunogenicity and are more suitable for subsequent structural optimization [36]. In order to obtain peptides with drug potential more effectively, we controlled the number of amino acids below 45. We screened NA39 for overlap by comparing it to a segment of RPL19, which is not the sequence that characterizes the toxin, but it is a sequence that encodes a large ribosomal subunit protein that is present in almost all species, is relatively conserved, and has no particular structure or properties. At the same time, NA39 is only a small segment of the RPL19 gene, and its structure and function are very different from the full protein characterized by RPL19, making it almost entirely novel. Given NA39’s ability to bind to TLR4, further studies may reveal a novel role for NA39 in the TLR4 pathway, which would be more relevant than determining whether it is derived from a toxin-associated sequence. The amino acid composition of NA39 contains 38.5% basic amino acids and 5.1% acidic amino acids. The results obtained from the server revealed that the multi-epitope vaccine contains alpha helix (74.4%) and coil (25.6%). The multi-epitope vaccine also contains small nonpolar (15.4%), hydrophobic (25.6%), polar (51.3%), and aromatics plus cysteine (7.7%) residues. Salt bridges are compounds formed by electrostatic interactions between charged ions [37]. The observation of amino acids in the binding site of NA39 and TLR4 revealed that the opposite charges in the multiple pairs of amino acids forming hydrogen bonds (T-379-ASP and N-13-LYS, T-453-ASP and N-5-HIS, T-502-ASP and N-4-ARG, T-287-GLU and N-20-LYS, T-405-ASP and N-13-LYS) are likely to form electrostatic interactions and further salt bridges, which may enhance intermolecular interactions [38] and contribute to the stability of the NA39-TLR4 complex, which is of great importance for the next role of NA39.

## 4. Conclusions

A phage display library was generated utilizing the venom gland of cobras in the present study, enabling the screening of various targets to identify precursor molecules that exhibit activity against diverse pathways. The screening process focused on TLR4 as the target, resulting in the identification of a 39-amino acid peptide named NA39. Molecular docking analysis was used to determine the potential binding sites between NA39 and TLR4, while SPR technology confirmed a robust binding interaction between TLR4 and NA39.

## 5. Materials and Methods

**Figure 6:**
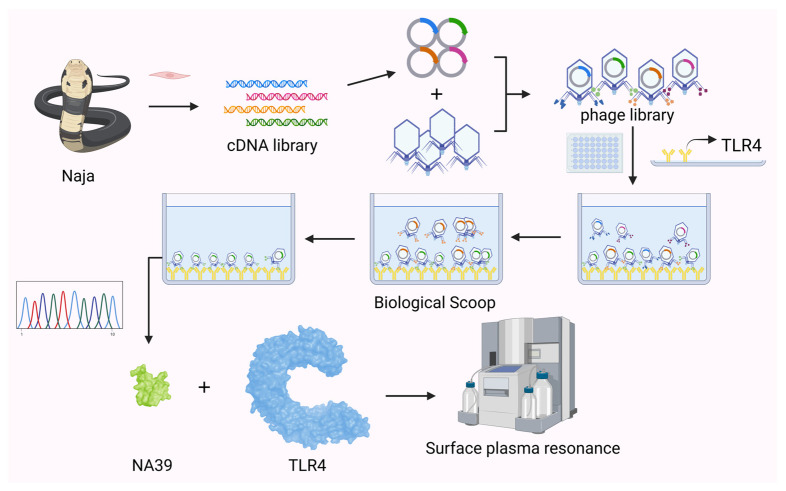
Experimental flow chart. The cobra venom gland was taken out to construct a cDNA library. After the cDNA was grafted into T7 arms, the packaging protein was added to construct a phage display library. TLR4 was coated on an ELISA plate, and the phage library was screened three times. The obtained sequence was analyzed, and peptide NA39 was selected for synthesis. Finally, the binding force between peptide NA39 and TLR4 was verified by SPR.

We conducted and experiment to construct a complete phage display and screening process and verified the screened outputs using SPR (Figure 6).

### 5.1. Construction of cDNA Library

In this study, the sample of *Naja atra* from the Guangdong Snake Farm was used to construct the library. The snakes were euthanized in closed containers with cotton-soaking isoflurane, and then the venom glands were removed after sacrifice and left in liquid nitrogen for 2 h before being transferred to −80 °C for long-term storage. For the extraction of total RNA, the venom glands were rapidly transferred to a mortar precooled with liquid nitrogen and then ground into a powder. The total RNA was extracted by RNAisoPlus (Takara, Beijing, China) and purified. Gel electrophoresis was immediately performed to verify the integrity of the extracted total RNA, and the concentration of the extracted total RNA was detected using Nanodrop (Thermo, Shanghai, China). Then, Poly (A) + mRNA was isolated from the total RNA with an Oligotex kit (QIAGEN, Beijing, China). The cDNA was reverse transcribed using the SMART^TM^ cDNA construction technique with reagents provided by Takara. An oligo (dT) sequence containing the Hind III cleavage site was synthesized from the 3′ polyA structure of the mRNA as the starting primer, designated SMART1—AAGCAGTGGTATCAACGCAGAGTGAATTCGGGG. An oligo (dG) sequence containing the EcoR I cleavage site was synthesized from the 5′ cap structure of the mRNA as an extension template for the first strand of the reverse transcription, designated CDS III primer—ATTCTAGACAAGCTTGGACATG-d(T)30 VN (N = A, G, C or T; V = A, G or C). The final first strand of cDNA obtained contained an enzymatic cleavage site at each end. The CDS Ⅲ primer was used as the 3′ PCR primer and synthesized the 5′ PCR primer—AAGCAGTGGTATCAACGCAGAGT for the second strand synthesis. After purification with the kit (Beyotime, Shanghai, China), the cDNA library of the cobra venom gland was generated, and the product was stored at −20 °C for an extended period of time.

### 5.2. Construction of Phage Display Library

According to manuals of the T7 Select Cloning Kit (Novagen, NJ, USA) and the T7 Select System, a T7 phage display library of the cobra toxin with BLT5403 as the host strain was constructed. In order to connect the cDNA with the arms of the T7 vector, the cDNA was digested with QuickCut™ EcoR I (Takara, Beijing, China) and QuickCut™ Hind III at 37 °C for 10 min to form the same sticky end as the T7 vector. After purification, the digested product was ligated with T7 select vector arms (Novagen) by T4 ligase (Takara, Beijing, China) and assembled into the T7 recombinant phage at 16 °C. The recombinant plasmid was packaged with T7 packaging extract (Novagen, Gibbstown, NJ, USA) to complete the construction of the phage display library.

To keep the phage display library highly active for a long time, it is necessary to amplify the original library. We added 300 μL of the original library into 5 mL BLT5403 bacterial solution with OD 0.8, mixed well, added 3 mL of top agar at 55 °C temperature into 1 mL of the mixture, turned it over and shook it quickly, mixed it well, and then and poured it into an LB Petri dish at 37 °C temperature. The culture was inverted in a 37 °C incubator for 3 h, which resulted in a large number of plaques. The following steps were conducted orderly: 10 mL of Phage Extraction Buffer was added to the Petri dish, which was placed at 4 °C overnight, and then the liquid was sucked from the Petri dish, 500 μL chloroform was added, the liquid was mixed well and centrifuged, and the supernatant was transferred to a new tube to obtain a phage amplification library. Then, 0.1 times the volume of 80% glycerol was added, and it was stored at −80 °C until use.

In order to check the titer of the library, 100μL was diluted into phage solutions of different concentrations: 250 μL BLT5403 bacterial solution with OD 0.8, and 3 mL of top agar kept at 55 °C were added. It was then quickly turned over and shaken, mixed evenly, poured into an LB Petri dish kept at 37 °C, and inverted cultured in a constant temperature incubator at 37 °C for 3–4 h. We took out all Petri dishes, counted them according to the plate with the least number of visible plaques, and used a dilution of 10 times as the titer of the phage library. We also provided the protocol for the construction of the phage display library (Supplemental File S3).

### 5.3. Biopanning

The TLR4 (Abcam, Shanghai, China) used in this study is a commercial protein sold by Abcam Company, which only contains the extracellular domain of the TLR4 protein with 24–632 amino acids. With TLR4 as the target, three rounds of biological panning of the phage library were carried out. TLR4 powder was centrifuged, redissolved with water to 500 μg/mL for storage, and diluted with PBS to 10 ng/μL for screening. The detailed procedures were as follows: the diluted protein solution was added to the blank enzyme-labeled plate, which was coated overnight at 4 °C, the plate was washed twice with TBST solution, and then detection buffer was added followed by incubation at room temperature for 2 h for blocking. After washing the plate twice with TBST solution, the phage amplified library was added to the plates, which were incubated at room temperature for 2 h. After washing the plate five times, Phage Extraction Buffer 100 μL was added at room temperature for 20 min. The collected Phage Extraction Buffer was mixed with BLT5403 fresh bacterial liquid with OD 0.5–0.6, and then the culture was shaken at 37 °C for amplification until the bacterial liquid became clear from a turbid state. After the culture, the phage liquid was centrifuged for 10 min, and the supernatant was taken. It was stored at −80 °C for a long time after adding 80% glycerol.

The output of the last screening was used as the input for the next screening, repeating three rounds of biological panning. The products of the third screening were diluted and cultured. The plates with clear phage clones were selected, and the monoclonal plaques were randomly selected for PCR. The primer sequences of PCR were as follows: T7 up, GGAGCTGTCGTATTCCAGTC; T7 down, AACCCCTCAAGACCCGTTTA. The PCR products were sequenced to obtain the insertion sequence of random plaque, that is, the polypeptide sequence that can bind to TLR4.

### 5.4. Molecular Docking Screening

PSIPRED 4 (Protein Structure Prediction Server) was used to predict the secondary structure of NA39 [24]. The tertiary structure of NA39 was modeled using SWISS-MODEL [25], and finally, the PDB file with the highest global model quality estimate (GMQE) score was selected for docking. The constructed model had a GMQE score of 0.91, and the referenced homologous structures were 7oyc.34.A 60S ribosomal protein L19 Cryo-EM structure of the Xenopus egg 80S ribosome. The obtained insertion sequence was input to the NCBI database for BLAST alignment to determine the source of the sequence. The base sequence obtained by sequencing was translated into an amino acid sequence by the Expasy-Translate tool, and with that, the sequence containing three or more stop codes was removed. The crystal structure of the TLR4 extracellular part was obtained from the PDB database, the number was 3fxi_A, and the inserted sequence was molecularly docked with the TLR4 structure by the HDOCK Server [39]. According to the docking score and confidence score, the sequence with a stronger binding force was selected. Protone-sol [40] and the Expasy-ProtParam tool [41] were used to predict the solubility and stability of the screened sequence, and the most potential sequence was selected for polypeptide synthesis by considering many indexes comprehensively.

### 5.5. Peptide Synthesis

First, Fmoc-AA-Wang resin preloaded with the first amino acid was added to the reactor, dimethyl formamide (DMF) was added to the immersed resin (Supplemental File S4), and swelling was carried out for 15 min. Then, 20% PiP/DMF solution was added to the reactor, it was washed twice to remove the Fmoc protective group, and then DMF was added to wash and remove PiP. A resin spot with a mixture of 150 µL Detection Reagent (6% ninhydrin in ethanol, 80% phenol in ethanol, and 2% 0.001 M KCN in pyridine solution) was put into one test tube and held at 100 °C for 20 s. The change in resin color served as an indicator of the successful removal of the Fmoc group. After adding the amino acid solution to the washed resin, 1 mL of di-isopropyl carbodiimide (DIC)/DMF solution was added and it was shaken for 1 h. The Kaiser test was used to confirm complete Fmoc-aa coupling: when no change in the color of the resin was observed, the amino acid coupling was confirmed to be successful. The operation was repeated until the target peptide was synthesized to the required length. After the synthesis, the crude sample solution was injected into the machine, and then the qualified fraction solutions were collected and freeze-dried by comparing with the purity requirements through HPLC analysis so as to obtain the freeze-dried powder of the polypeptide. The secondary structure of NA39 was detected by CD. The dry powder of NA39 was dissolved in ultrapure water, and the concentration was diluted to 0.067 mg/mL as the test sample. After switching on the instrument, the parameters were set as required, and the detection wavelength range was set to 180–260 nm. The air spectrum was detected first, then the ultrapure water used to dissolve NA39 was added to the cuvette for the blank group detection, and finally, the sample at the concentration of 0.067 mg/mL was added for detection. Each group of detections was repeated three times, with the cuvette placed in the same direction for each detection. Raw data were converted to Δε after pre-processing, such as averaging, baseline subtraction, smoothing, etc. The protein secondary structure analysis was performed on the online website DichroWeb “http://dichroweb.cryst.bbk.ac.uk/html (accessed on 16 January 2024)” [42] using the CDSSTR method [43] and the SMP180t dataset [44]. The scaling factor was set to 1.0. and the results were fitted. The corresponding data are provided in Supplemental File S5.

### 5.6. SPR Verification of the Binding Force between NA39 and the TLR4 Protein

The binding of peptide NA39 with the TLR4 extracellular part (24–632aa) (Abcam) was verified by the molecular interaction detection instrument Biacore T200 (Uppsala, Sweden). The TLR4 was consistent with the biopanning test, according to the formula:

(2) $$R_{max}=\frac{analyteMW}{ligandMW}\times R_{L}\times S_{m}$$

The target protein coupling amount was calculated. In the formula, R_max_ is the maximum binding capacity on the chip surface, and 100 RU is generally substituted in protein inspection. Analyte MW and ligand MW are the molecular weight of TLR4 and peptide NA39, respectively. S_m_ is the stoichiometric ratio, usually being 1, and RL is the ligand coupling level. The actual coupling amount is usually 1.5 times R_L_. The buffer used during the experiments was 1× HBS-EP + buffer (10×, 0.1 M, HEPES; 1.5 M, Nacl; 0.03 M, EDTA; 0.5%, P20), and all reagents needed to be filtered with 0.22 filter membrane before use. The TLR4 protein powder underwent high-speed centrifugation, was reconstituted in dd water to form a 400 µg/mL mother liquor for future use, and then was subsequently diluted to a 20 µg/mL concentration using sodium acetate at pH levels of 4.0, 4.5, and 5.0. Screening of protein coupling conditions was conducted, and the results indicated that pH 4.0 provided the optimal conditions for TLR4 coupling. The maximum response concentration was 2500 mΜ. To initiate coupling, first, the chip was activated by mixing 1-(3-Dimethylaminopropyl)-3-ethylcarbodiimide hydro (EDC) and N-Hydroxysuccinimide (NHS) (GE Healthcare) in equal amounts. Then, it was coupled with a protein solution, which was diluted with sodium acetate at pH 4.0, and the coupling amount was set to 2206.55 RU. After the coupling was completed, the chip surface was sealed with ethanolamine (GE Healthcare). The instrument automatically generated a result after the coupling was completed, and Target Reached displayed “Yes” to indicate a successful coupling. At the end, a channel in CM5 chip was used as a blank reference, without TLR4, and the rest were treated identically. After successful protein coupling, the regeneration conditions were screened. The screening reagent used was Glycine-HCl solution at 10 mM, pH 2.0, 2.5, and 3.0, and the complete regeneration reagent was determined to be glycine–HCl, pH 2.0, based on the baseline changes shown in the experimental results.

Peptide NA39 analytical solutions with different concentrations were prepared according to the gradient, which were then flowed through the chip coupled with TLR4 in sequence from low to high. After binding, the chip was regenerated with the glycine–HCl solution at pH 2.0, and each concentration was repeated three times. The response of the experimental channel minus that of the blank channel was the result of the binding force between peptide NA39 and TLR4. The data output by SPR was directly processed by BIA evaluation 3.2 software. We also provided the sensorgrams corresponding to the coupling of TLR4 to the surface and the regeneration condition (Supplemental File S6).

## Figures and Tables

**Table 1:** Prediction results of the top 10 amino acid sequences.

Number	Amino Acid Number	Binding Scores	Solubility	Instability	Pl
1	52	0.9769	0.686	34.73	11.68
2	39	0.9172	0.701	39.85	11.17
3	67	0.9261	0.566	63.32	9.34
4	68	0.9663	0.579	55.88	11.92
5	55	0.9556	0.642	63.63	12.44
6	65	0.9393	0.575	51.99	10.44
7	38	0.9331	0.575	53.18	11.58
8	43	0.9306	0.567	46.01	12.33
9	33	0.9239	0.679	50.15	12.63
10	28	0.919	0.606	30.13	7.15

## Data Availability

Data is contained within the article.

## Supplementary Materials

The following supporting information can be downloaded at: https://www.mdpi.com/article/10.3390/toxins16030113/s1, Supplemental Files S1–S6.
