# Supplementary material for: Screening TLR4 Binding Peptide from Naja atra Venom Glands Based on Phage Display
Source: Toxins (Basel). 2024 Feb 24;16(3):113. doi: 10.3390/toxins16030113 (PMC10976260; doi:10.3390/toxins16030113)
Supplement: Supplementary file 1 [file toxins-16-00113-s001.zip › Supplemental file S3.pdf]

Construction of phage display library Protocol

1 SMART® cDNA Library Construction

1.1 Introduction

The SMART cDNA Library Construction provides a method for producing high-quality, full-length cDNA libraries from nanograms of total or poly A+ RNA. The methods utilize the proprietary SMART IV (Switching Mechanism At 5' end of RNA Template). Oligonucleotide in the first-strand synthesis to generate high yields of full-length, double-stranded (ds) cDNA (Figure S1).

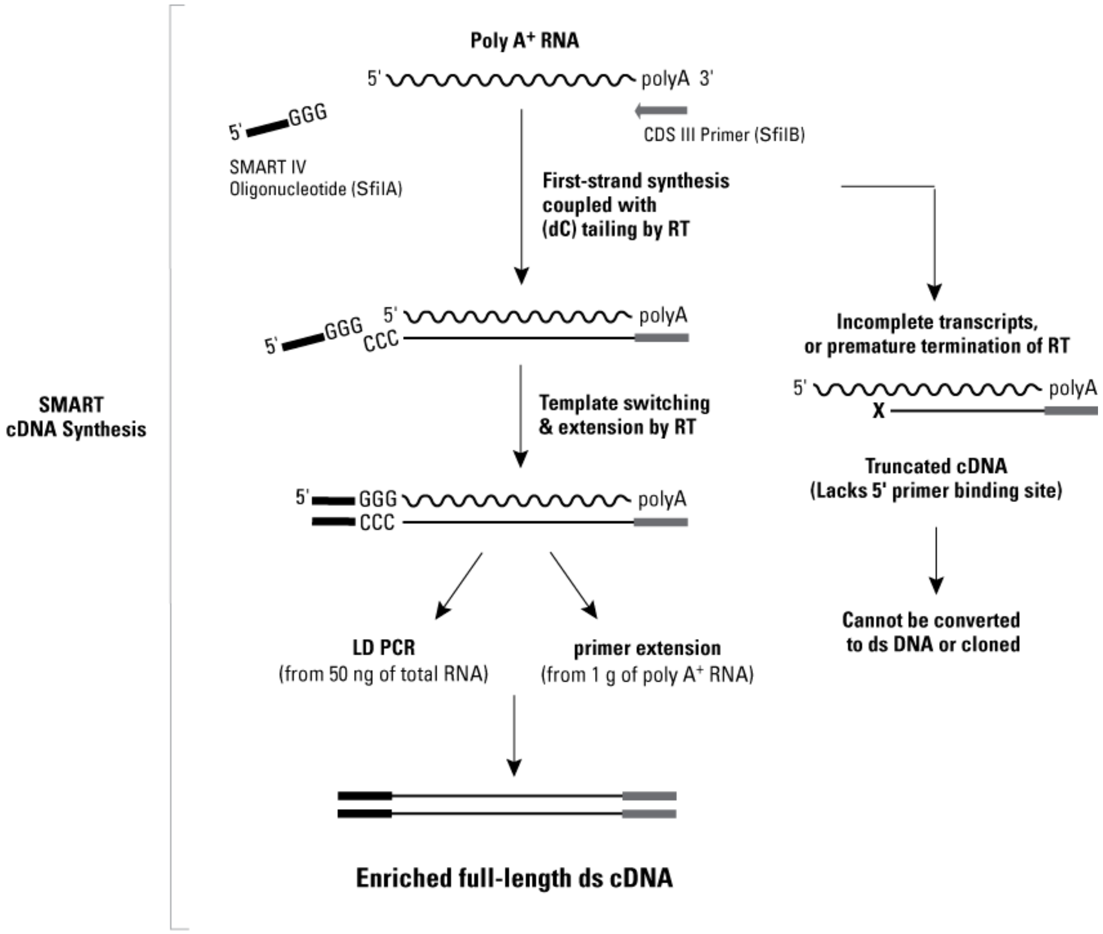

Figure S1. Flow chart of the SMART cDNA Library Construction protocols. The right side of the flow chart shows the fate of incomplete transcripts caused by RNA degradation or premature termination of reverse transcription.

1.2 First Strand cDNA Synthesis

Combine the following components for experimental RNA sample and a positive control in separate sterile 0.5 ml microcentrifuge tubes as follows,

	Reagent Volume(μl per sample)	
	**Experimental RNA Sample**	**Positive Control RNA Sample**
**Poly A+RNA(0.025-0.5μg)**	3 μl	–
**Control Poly A+RNA(1.0 μg)**	–	1 μl
**SMART IV Oligonucleotide**	1 μl	1 μl
**CDS /3'PCR Primer**	1 μl	1 μl
**Deionized H_2O**	0 μl	2 μl
Total Volume	5 μl	5 μl

Mix contents and spin the tube briefly in a microcentrifuge.

Incubate the tube at 72°C for 2 min.

Cool the tube on ice for 2 min.

Spin the tube briefly to collect the contents at the bottom.

Add the following reagents to each reaction tube which already contains 5 μl from Step 1

2.0 μl	5X First-Strand Buffer
1.0 μl	DTT (20 mM)
1.0 μl	dNTP Mix (10 mM)
1.0 μl	SMARTScribe MMLV Reverse Transcriptase
10.0 μl	Total Volume

Mix the contents of the tube by gently pipetting and briefly spinning the tube.

Incubate the tube at 42°C for 1 hr in an air incubator or a hot lid thermal cycler.

Place the tube on ice to terminate first-strand synthesis and proceed as follows.

Any first-strand reaction mixture that is not used right away should be placed at –20°C. First-strand cDNA can be stored at –20°C for up to three months.

1.3 ds cDNA Synthesis by LD PCR

Preheat the thermal cycler to 95°C.

Combine the following components:

2 μl	First-Strand cDNA
80 μl	Deionized H_2O
10 μl	10X Advantage 2 PCR Buffer
2 μl	50X dNTP Mix
2 μl	5' PCR Primer
2 μl	CDS III/3' PCR Primer
2 μl	50X Advantage 2 Polymerase Mix
100 μl	Total volume

Mix contents by gently flicking the tube. Centrifuge briefly to collect the contents at the

bottom of the tube.

Cap the tube and place it in a preheated (95°C) thermal cycler.

Perform LD-PCR using the following programs:

Hot-lid thermal cycler:

95°C for 20 sec

25cycles: 95°C for 5 sec , 68°C for 6 min

When the LD-PCR reaction is complete, analyze a 5 μl sample on a 1.1% agarose/EtBr gel, and store ds cDNA at –20°C until use

2 **Construction of phage display library**

2.1 Enzyme digestion

Combine the following components in a fresh 1.5 ml tube:

3 μl	DNA
5 μl	10X QuickCut Buffer
1 μl	QuickCut™ EcoR I
1 μl	QuickCut™ Hind III
40μl	Deionized H2O
100 μl	Total volume

Mix well. Incubate the tube at 37°C for 5 min

2.2 Ligation of Inserts and Vector Arms

Set up ligation reactions by assembling the following components in a sterile 0.5 ml or 1.5 ml tube (add ligase last):

2.5 μl	DNA
1.0 μl	T7Select® Vector Arms
0.5 μl	10X Ligase Buffe
1.0 μl	T4 DNA Ligase
5 μl	Total volume

Gently pipet up and down and then incubate 3–16 h at 16°C. Store at 4°C until use.

2.3 In Vitro Packaging

Allow the T7Select® Packaging Extract to thaw on ice. The volume of the extract is 25 μl and will package up to 1 μg of vector DNA without a loss in efficiency.

Add 5 μl ligation reaction per 25 μl extract. Mix gently by stirring with a pipet tip; do not vortex. To test the packaging efficiency independently, add 0.5 μg of the control DNA to 25 μl extract.

Incubate the reaction at room temperature (22°C) for 2 h.

Stop the reaction by adding 270 μl sterile LB or TB medium. If the packaging reaction will be stored for more than 24 h prior to amplification, add 20 μl chloroform and mix gently by inversion. The packaging reaction can be stored for up to one week at 4°C without significant losses in titer. For longer term storage, the packaged phage must be amplified by

plate or liquid culture methods.

When using the T7Select System, packaged libraries must be amplified by either the plate or liquid culture methods prior to biopanning. Amplification is necessary for the expression of cloned sequences and their display on the surface of phage particles.

2.4 Amplifying Libraries

Inoculate 50 ml LB with 1 ml overnight culture of the appropriate host(BLT5403) . Shake at 37°C until the OD600 reaches 0.6–1.0.

Mix the phage (packaging reaction) with 5ml host in a sterile 15 ml tube.

Transfer 1 ml aliquots of the phage/host mixture into sterile 15 ml tubes.

Add 3 ml molten top agarose at 45–50°C to each tube. Immediately pour the contents of the tube onto a 150 mm LB Amp plate.

Spread the top agarose evenly by gently swirling the plate.

Allow the plates to sit undisturbed on a level surface so the top agarose can solidify. Invert the plates and incubate in 37℃ until the plaques are nearly confluent.

To elute the phage, cover each plate with 10 ml of Phage Extraction Buffer (20 mM Tris-HCl, pH 8.0, 100 mM NaCl, 6 mM $MgSO_4$) and place on a level surface at 4°C from 2 h .

Harvest the phage by tipping the plate slightly and pipetting the liquid into a sterile container. Combine the extract buffer from all the plates in a single tube or bottle. Add 0.5 ml chloroform and gently mix. Centrifuge at 3,000 × g for 5 min to clarify the lysate and transfer the supernatant to a sterile tube or bottle.

Determine the titer of the amplified library by plaque assay. Amplified library lysates can be stored at 4°C for several months without a loss of titer. For longer term storage, add 0.1 volume sterile 80% glycerol and store at –70°C.
