# Supplementary material for: Screening TLR4 Binding Peptide from Naja atra Venom Glands Based on Phage Display
Source: Toxins (Basel). 2024 Feb 24;16(3):113. doi: 10.3390/toxins16030113 (PMC10976260; doi:10.3390/toxins16030113)
Supplement: Supplementary file 1 [file toxins-16-00113-s001.zip › Supplemental file S6.pdf]

TLR4 Coupling

G:\??2023.8.30?\lrh-1\lrh result23.05.18.blr

Instr. id: 2394880 Biacore T200 Control Software, version 3.2.1 Print date: 1/9/2024 3:19:04 PM

Immobilization Wizard results

Chip: CM5
Flow cells per cycle: 1

Flow cell	Procedure	Method	Ligand	Response Bound (RU)	Response Final (RU)	Target Reached
4	Target level	Amine	TLR4	2290.6	2136.6	Yes

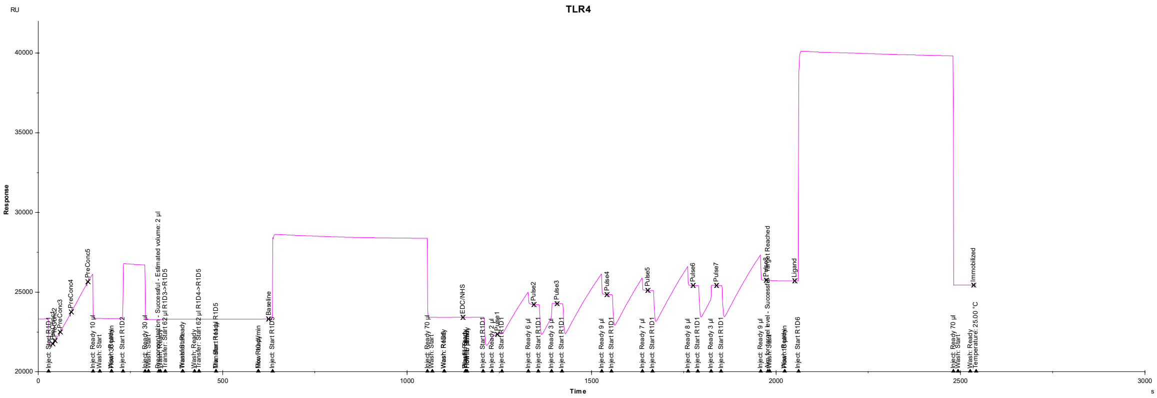

Regeneration Condition

G:\??2023.8.30?\lrh-1\ph result lrh 23.05.18.blr

Instr. id: 2394880 Biacore T200 Control Software, version 3.2.1 Print date: 1/9/2024 3:25:17 PM

Regeneration scouting result

Trend Chart

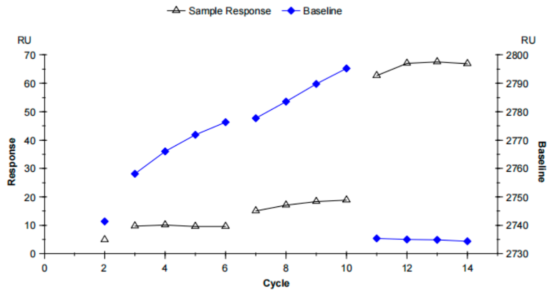

Subtracted Fc=4-3

Selected conditions

Condition 1 Solution: Glycine-Hcl3.0, 30(s)
Condition 2 Solution: Glycine-Hcl2.5, 30(s)
Condition 3 Solution: Glycine-Hcl2.0, 30(s)

Sensorgrams

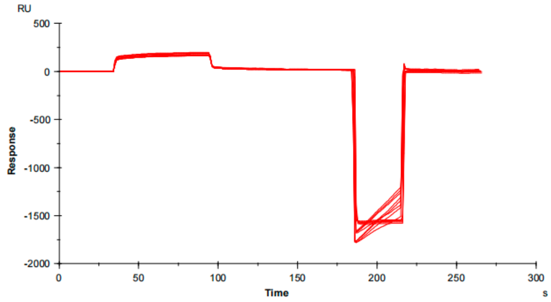

Sensorgram Fc=3

Included cycles for each condition: 1, 2, 3, 4

Selected conditions

Condition 1 Solution: Glycine-Hcl3.0, 30(s)
Condition 2 Solution: Glycine-Hcl2.5, 30(s)
Condition 3 Solution: Glycine-Hcl2.0, 30(s)

TLR4-NA39 Kinetic Raw Sensorgram

Path 4-3

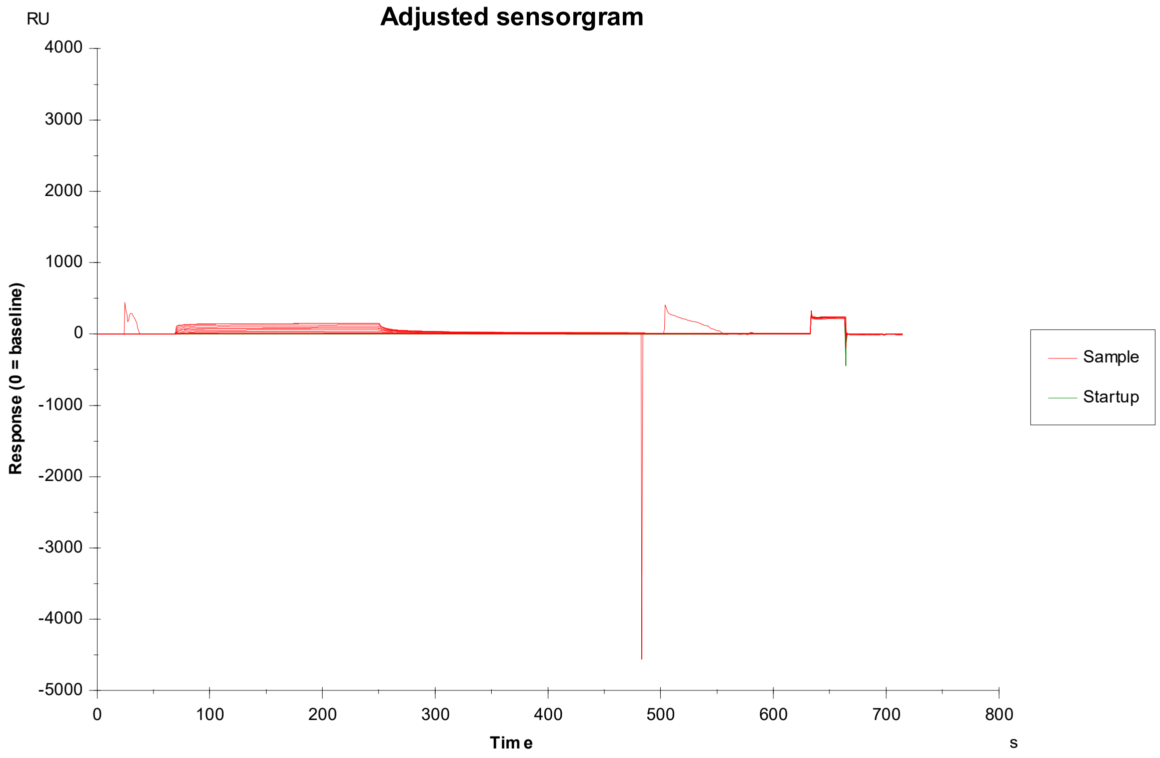

Path 3 Blank Control group

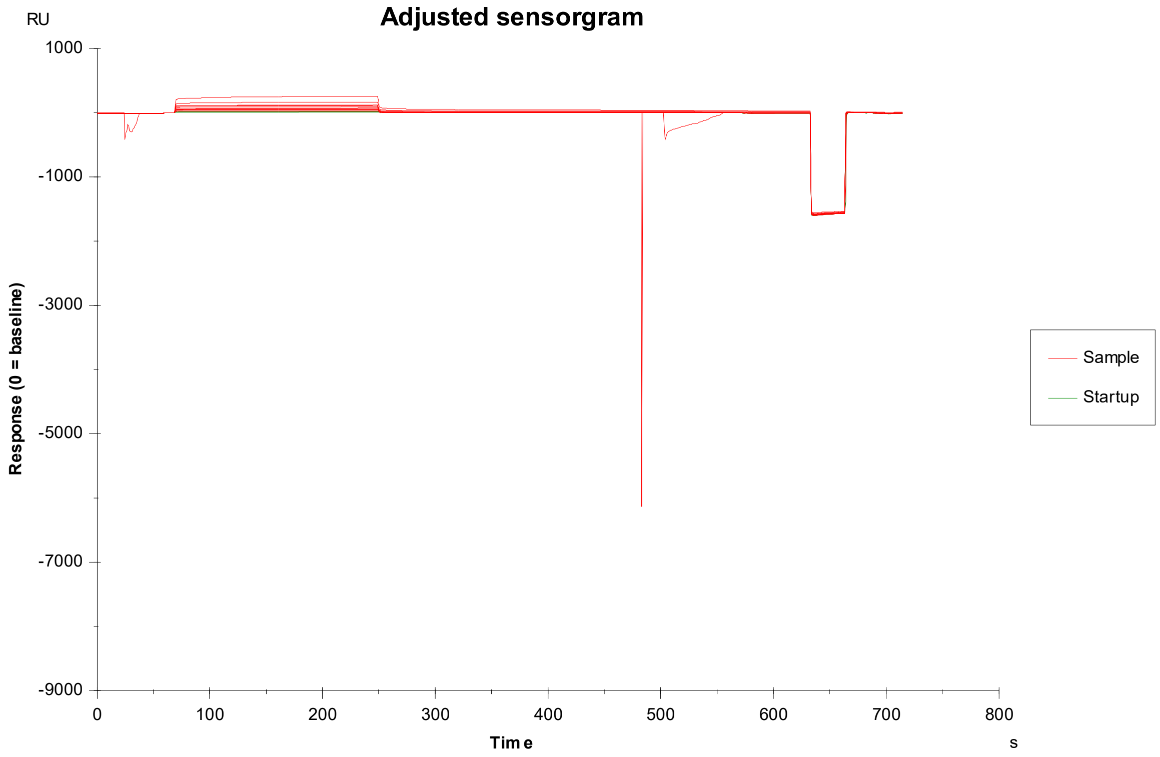

Path 4 Experimental Group

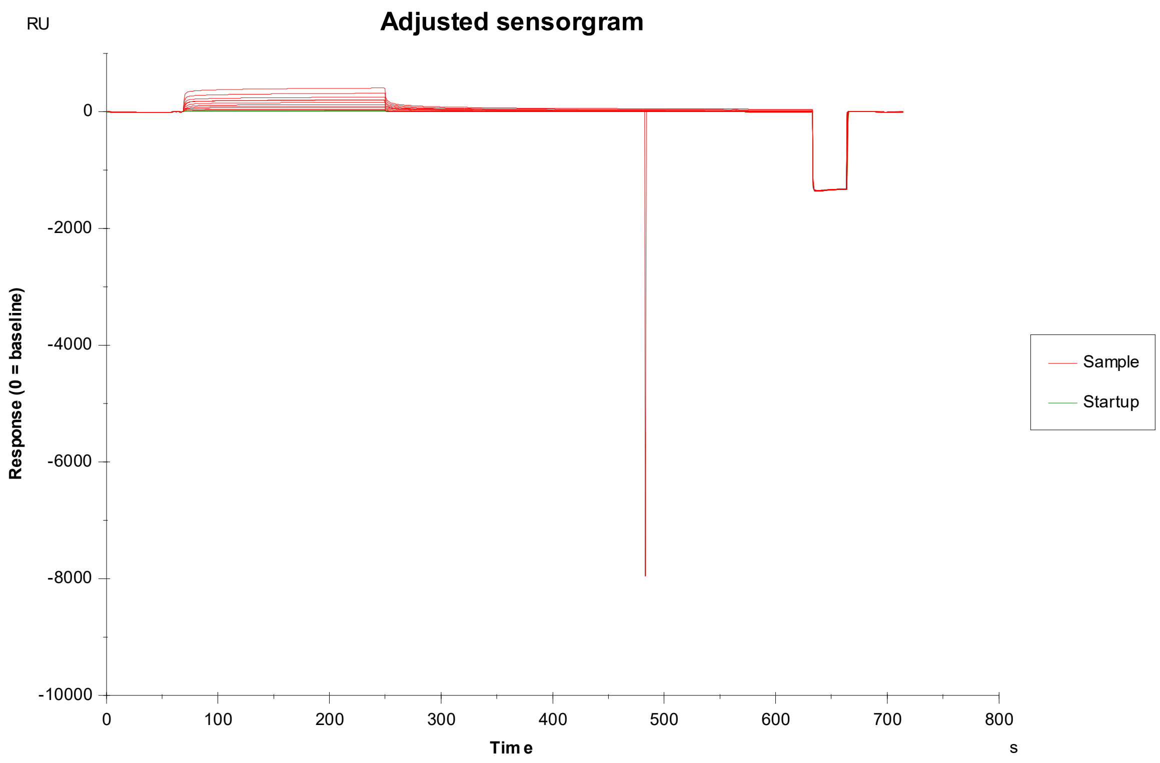

The regeneration time between 600-700s
